# CXCR7 signaling promotes breast cancer survival in response to mesenchymal stromal stem cell-derived factors

**DOI:** 10.1038/s41420-019-0169-3

**Published:** 2019-04-11

**Authors:** Mashael Al-toub, Mohammad Almohawes, Radhakrishnan Vishnubalaji, Musaad Alfayez, Abdullah Aldahmash, Moustapha Kassem, Nehad M. Alajez

**Affiliations:** 1 Stem Cell Unit, Department of Anatomy, College of Medicine, King Saud University, Riyadh, 11461 Saudi Arabia; 2 College of Applied Medical Sciences, King Saud University, Riyadh, 11461 Saudi Arabia; 3 Cancer Research Center, Qatar Biomedical Research Institute (QBRI), Hamad Bin Khalifa University (HBKU), Qatar Foundation (QF), PO Box 34110, Doha, Qatar; 4 Prince Naif Health Research Center, King Saud University, Riyadh, 11461 Saudi Arabia; 5 Molecular Endocrinology Unit (KMEB), Department of Endocrinology, University Hospital of Odense and University of Southern Denmark, Odense, Denmark; 6 Department of Cellular and Molecular Medicine, Danish Stem Cell Center (DanStem), University of Copenhagen, 2200 Copenhagen, Denmark

## Abstract

The interaction between cancer cells and molecular cues provided by tumor stromal cells plays a crucial role in cancer growth and progression. We have recently reported that the outcome of interaction between tumor cells and stromal cells is dependent on the gene expression signature of tumor cells. In the current study, we observed that several cancer cell lines, e.g., MCF7 breast cancer line, exhibited growth advantage when cultured in the presence of conditioned media (CM) derived from human bone marrow stromal stem cells (hBMSCs). Regarding the underlying molecular mechanism, we have identified CXCR7 as highly expressed by MCF7 cells and that it mediated the enhanced growth in response to hBMSC CM. Regarding the clinical relevance, we found an inverse correlation between the level of tumor gene expression of CXCR7 in bladder, breast, cervical, kidney, liver, lung, pancreatic, stomach, and uterine cancers, and patients’ overall survival. Interestingly, significant positive correlation between CXCR7 and CXCL12 gene expression (Pearson = 0.3, *p* = 2.0 × 10^–16^) was observed in breast cancer patients, suggesting a biological role for the CXCR7/CXCL12 genetic circuit in breast cancer biology. Our data provide insight into the molecular mechanisms by which stromal-derived microenvironmental cues mediate CXCR7 signaling and growth enhancement of breast cancer cells. Therapeutic targeting of this circuit might provide novel therapeutic opportunity for breast cancer.

## Introduction

Carcinogenesis is a complex process resulting from an interplay between malignant cells and microenvironmental cues, including extracellular matrix, endothelial cells, pericytes, immune infiltrating cells, and carcinoma-associated fibroblasts (CAFs)^1^. This interaction contributes to tumor growth, invasion, and metastasis. Among the tumor microenvironment components, the role of CAFs in cancer development is an area of intensive investigation. A number of studies have suggested that CAFs are derived from mesenchymal (stromal) stem cells (MSCs), which are multipotent stem cells present within the stroma of bone marrow and other organs^2^. The precise role of CAFs or MSCs in cancer development and progression remains controversial^3,4^. Our recent experimental studies suggested reciprocal interaction between cancer cells and MSCs^5,6^. In a co-culture system, we observed that the effects of human bone marrow-derived MSCs (hBMSCs) on cancer cells were largely dependent on the expression of IL1β and CDH1 by tumor cells^6^. However, the exact molecular factors secreted by hBMSCs and their cognate receptors that promote cancer growth remained largely unknown.

Chemokines and their receptors represent a family of signaling molecules that may play a role in breast cancer progression. Karnoub et al. reported a role for BMSC-derived C-C Motif Chemokine Ligand 5 in promoting breast cancer metastasis to the lung^7^. Similarly, secretion of stromal cell-derived factor 1 (SDF1, also known as C-X-C Motif Chemokine Ligand 12 (CXCL12)) by BMSCs has been implicated in bone metastases of breast cancer cells^8^. However, there are limited data on the effect of hBMSCs-derived factors on tumor growth. Herein, we performed a systematic approach to assess the role of secreted factors from hBMSCs in tumor growth in vitro and identified C-X-C Chemokine Receptor Type 7 (CXCR7) expressed by tumor cells as a key receptor driving cancer cell proliferation and survival.

## Results

### Effect of hBMSC-conditioned media (MCM) on in vitro cancer cell growth

**Fig. 1:**
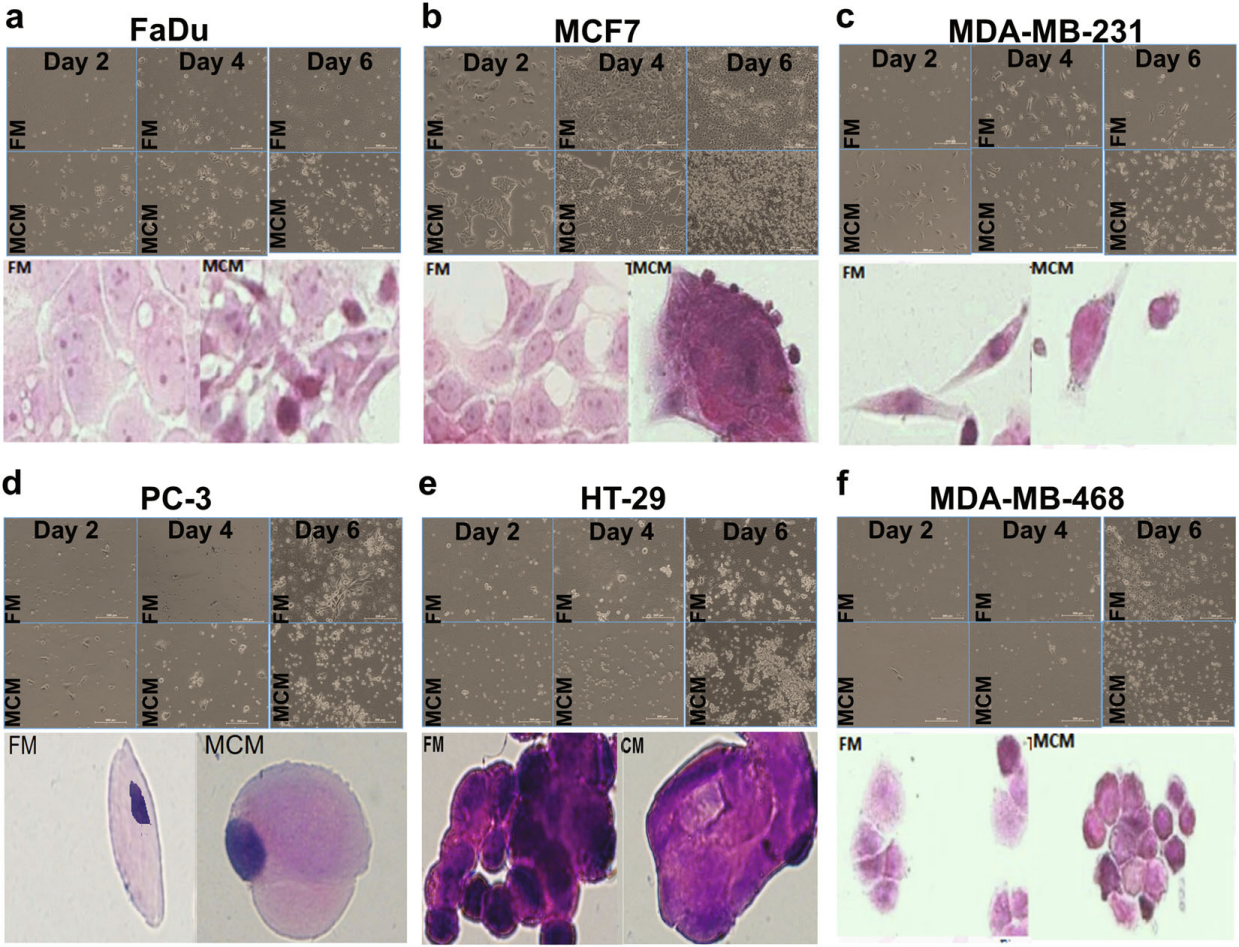
Effect of hBMSC-derived conditioned media on tumor growth. Bright field (upper panels) images of FaDu, MCF7, MDA-MB-231, PC-3, HT-29, and MDA-MB-468 tumor cell lines cultured in MCM or FM for 2, 4, or 6 days (10×). Representative image of H and E-stained tumor cells cultured in MCM or FM (lower panels). Images were taken on day 6 (20× (left) and 60× (right) panel). MCM human bone marrow stromal stem cells conditioned media, FM fresh media

To assess the effect of MCM on tumor cell growth, tumor cell lines were cultured (day 0) in the presence of MCM control fresh medium (FM). Both MCM and FM were supplemented with 1% fetal bovine serum (FBS). We examined cell growth in cancer cells on day 2, day 4, and day 6 post exposure. Qualitative analysis revealed tumor cells cultured in MCM exhibited enhanced cell proliferation and reached confluency at an earlier time point compared to cells cultured in FM (Fig. 1). In particular, MCF7 exhibited the most pronounced response (Fig. 1b).

### Secreted factors from hBMSC exhibited variable effects on different tumor cell lines

**Fig. 2:**
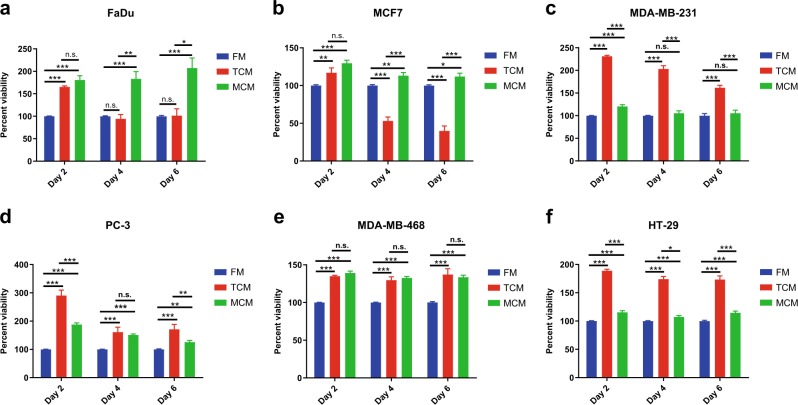
Effect of hBMSC-derived conditioned media on tumor viability. FaDu, MCF7, MDA-MB-231, PC-3, HT-29, and MDA-MB-468 tumor cells were cultured in FM, TCM, or MCM for 2, 4, and 6 days. Cell viability on the indicated days was assessed using alamarBlue. Data are presented as mean ± S.E.M. from a minimum of three experiments, *n* ≥ 20. *P ≤ 0.05, **P ≤ 0.005, ***P ≤ 0.0005. *p* values were calculated using two-tailed Student test with equal variance. Black bars indicate compared experimental groups. MCM human bone marrow stromal stem cells conditioned media, TCM tumor-derived conditioned media, FM fresh media

**Fig. 3:**
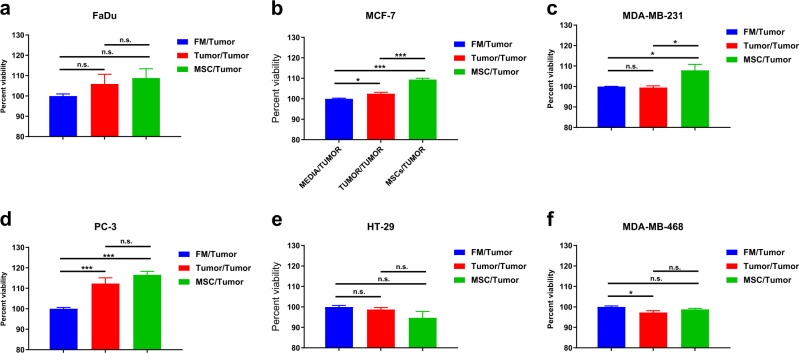
The effect of secreted factors from hBMSCs on tumor growth using the co-culture system. Cell viability of the indicated tumor cell line cultured under different experimental conditions using the transwell system (0.4 µm). Tumor cells were cultured in the lower chamber, while the other treatment was in the upper chamber. Cell viability was assessed using alamarBlue assay on day 6. Data are presented as mean ± S.E.M. from a minimum of three experiments, *n* ≥ 40. *P ≤ 0.05, **P ≤0.005, ***P≤0.0005. *p* values were calculated using two-tailed Student test with equal variance. Black bars indicate compared experimental groups

To rule out the possibility that the observed enhanced cell proliferation is mediated by general nonspecific factors released by mammalian cells, cancer cells were cultured under three different conditions: (i) in the presence of MCM derived from hBMSC, (ii) in the presence of own conditioned media (CM) (tumor conditioned medium) (TCM), or (iii) under FM. Cell viability was assessed using the alamarBlue assay on days 2, 4, and 6. As shown in Fig. 2, MDA-MB-231, PC-3, and HT-29 grew better when cultured in their own TCM (Fig. 2a–e). Although statistically significant, the effect of MCM on the growth of MDA-MB-231 and HT-29 on day 2 was modest. MDA-MB-468 grew equally well in their own TCM as compared to MCM (Fig. 2e). Interestingly, MCF7, and to a lesser extent FaDu on days 4 and 6, grew better in MCM compared to own TCM (Fig. 2a, b). Overall, all cell lines grew better in MCM compared to FM. In order to confirm these findings, we employed the co-culture trans-well assay experiment, where we cultured tumor cells in the lower chamber and hBMSC, tumor cells, or just media in the upper chamber. Tumor cells were included in the upper chamber in each experimental group to compensate for the total number of cells and to eliminate possible effects of general factors released by mammalian cells in a nonspecific manner. The two chambers are separated by a semipermeable membrane (pore size 0.4 µm) which allows only small molecules to pass through. MCF7 and MDA-MB-231 grew better under MCM condition (Fig. 3b, c), while PC-3 grew equally under their own TCM or MCM (Fig. 3d). No promoting effects of TCM or MCM on the growth of HT-29 and MDA-MB-468 were observed (Fig. 3e, f), while a modest growth-promoting effect of TCM and MCM was observed on FaDu cells, although it was not statistically significant (Fig. 3a). Taken together, our data revealed general promoting effects of MCM on the growth of MCF7, MDA-MB-231, PC-3, and FaDu cells.

### CXCR7 plays an important role in mediating the promoting effects of hBMSCs on MCF7 cells

**Fig. 4:**
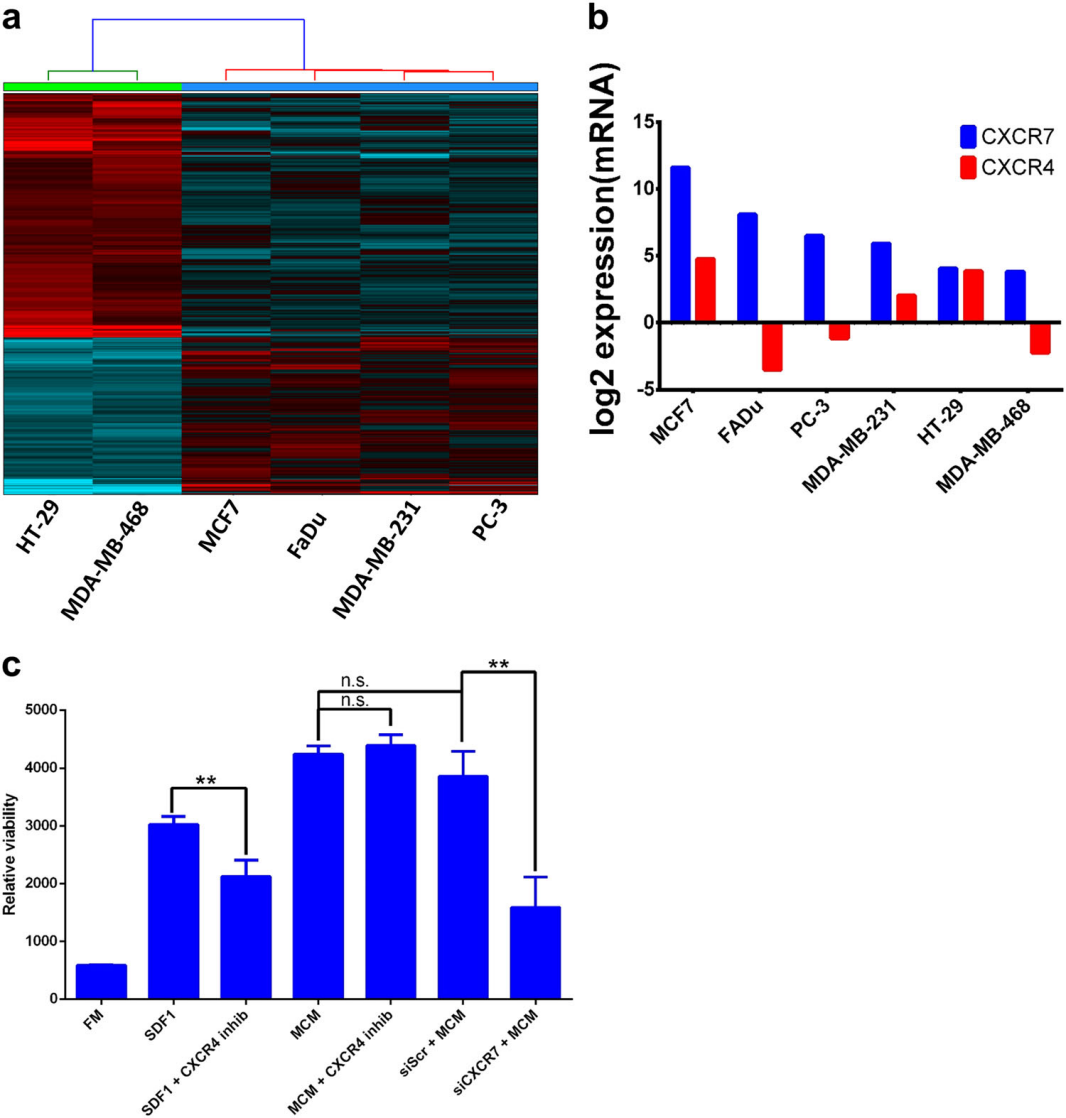
Gene expression analysis of tumor cell lines as a function of response to hBMSC-derived CM. **a** Hierarchical clustering based on differentially expressed genes between tumor cell lines that exhibited growth advantage (MCF7, FaDu, MDA-MB-231, and PC-3) compared to those that did not exhibit growth advantage (HT-29 and MDA-MB-468). **b** Bar chart depicting the expression of CXCR7 and CXCR4 on the indicated tumor cell lines. **c** Effect of inhibition of CXCR4 (using WZ811) or inhibition of CXCR7 on tumor cell growth in the presence of recombinant CXCL12 (SDF1) or hBMSC-derived CM. Data are presented as mean ± S.E.M. from three experiments

In order to identify potential surface receptors expressed on tumor cells that mediated the growth enhancement effects of MCM, we compared molecular signatures obtained from global gene expression analysis, between the tumor cell lines that were responsive to MCM (MCF7, FaDu, MDA-MB-231, and PC-3) and the nonresponsive cell lines (HT-29 and MDA-MB-468). Hierarchical clustering based on differentially expressed genes between the two groups is depicted in Fig. 4a. The top 100 upregulated genes in the responder group are shown in Supplementary Table 1. Interestingly, we observed that CXCR7 was upregulated >16.0 folds in the responder group compared to the nonresponders group. CXCR7, also known as ACKR3, is a chemokine receptor that binds to CXCL11 and CXCL12 (SDF1), while CXCR4 homodimer binds only to CXCL12^9^. Expression of CXCR7, but not CXCR4, correlated with the cancer cell response to MCM (Fig. 4b).

**Fig. 5:**
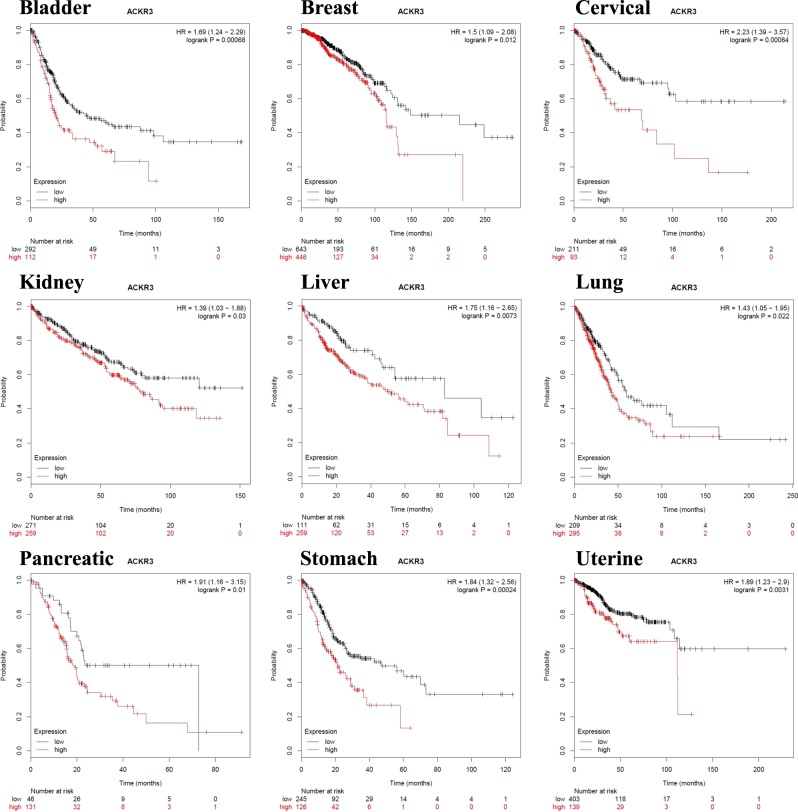
Expression of CXCR7 is associated with poor prognosis in several cancer types. Kaplan–Meier plots illustrate the duration of overall survival according to the expression of CXCR7 in bladder, breast, cervical, kidney, liver, lung, pancreatic, stomach, and uterine cancer. Log-rank test was used for curve comparison

**Fig. 6:**
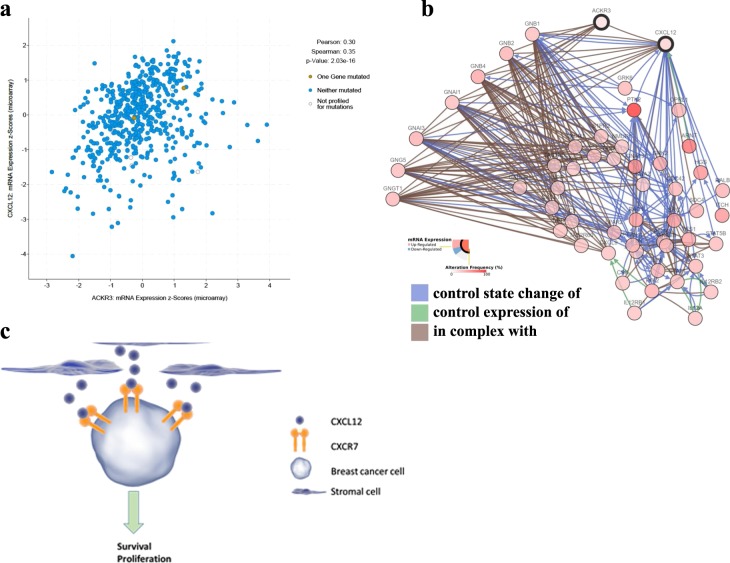
CXCR7 and CXCL12-dependent network interactions in breast cancer. **a** Scatter plot depicting the correlation between CXCR7 and CXCL12 expression in breast cancer. Pearson and Spearman correlations and associated *p* values are indicated. **b** Network analysis illustrating the interaction between CXCL12, CXCR7, and various members of the G-protein family. **c** Schema illustrating CXCL12 released by stromal cell to promote tumor cell survival and proliferation via binding to CXCR7 on breast cancer cells

Previous studies have suggested a role for SDF1/CXCL12 and its receptor CXCR4 in regulating cell migration and survival^10^, and a role for CXCR7 in mediating cancer tumor survival, and development^11^. Thus, we investigated the role of CXCR7 signaling in promoting tumor cell survival. Since MCF7 expressed the highest levels of CXCR7 (Fig. 4b), it was employed in the subsequent experiments. Incubating MCF7 with exogenous CXCL12 (SDF1) promoted cell growth and these effects were partially abolished by cotreatment with CXCR4 (WZ811) small-molecule inhibitor (Fig. 4c). Interestingly, MCM promoted MCF7 proliferation, which was not affected by CXCR4 inhibition (Fig. 4b). siRNA-mediated inhibition of CXCR7 expression diminished the growth enhancement effect of MCM, suggesting that signaling via CXCR7 is a regulatory mechanism promoting MCF7 growth in response to secreted factors present within MCM. To determine the clinical relevance of our observations, interrogation of the expression of CXCR7 in bladder, breast, cervical, kidney, liver, lung, pancreatic, stomach, and uterine cancers revealed significant poor overall survival in patients with tumors exhibiting elevated gene expression levels of CXCR7 (Fig. 5). Network analysis on the cancer genome atlas (TCGA) breast cancer dataset revealed interaction between CXCL12 and CXCR7 (ACKR3), and a number of G-protein family members (GNG5, GNB4, GNB2, GNG12, GNG7, GNGT1, and GNAI3, Fig. 6a). Significant correlation between CXCR7 and CXCL12 was also observed in the same patient cohort, suggesting a regulatory role for CXCR7 and CXCL12 in breast cancer biology (Fig. 6b). Schema depicting the role of hBMSCs in promoting tumor cells via CXCR7 signaling is illustrated in Fig. 6c.

## Discussion

Identification of factors regulating the interaction between cancer cells and microenvironment is an area of intensive investigation as it provides understanding of cancer development as well as targets for therapy. In this paper, we have demonstrated that factors secreted by stromal cells regulate cell proliferation of cancer cells and identified signaling through chemokine receptor CXCR7 as a possible mechanism.

Malignant tumors are composed of a multitude of cell types in addition to the malignant cells, and that include stromal cells, also known cancer-associated fibroblasts. Conflicting data exist in the literature regarding the precise role of stromal cells in cancer development and progression^7,12^. We have previously proposed that the discrepancy observed in the published results may be explained by differences in cancer type, as we observed that the interaction between tumor cells and stromal cells is dependent on the gene expression signature of tumor cells. In addition, we have identified IL1β and CDH1 expression by tumor cells as predictive markers for this interaction^5,6^.

We observed that cancer cell lines vary in the degree of responsiveness in terms of enhanced cell proliferation when exposed to CM derived from stromal cells. Interestingly, MCF7 breast cancer cell line exhibited the most pronounced response. These data corroborate our previous observations demonstrating increased cell growth of MCF7 cells when cocultured with hBMSCs in vitro^6^. Our data suggest that cancer cell–stromal cell interaction is cell type specific and dependent not only on the factors secreted by stromal cells, but also on the biological characteristics of cancer cells.

In order to identify the possible mechanism for differences in responsiveness to conditioned medium of stromal cells between different cancer cell types, we examined the molecular signature of cancer cells by using global gene expression analysis. We identified CXCR7 as highly expressed in MCF7 cells and as a possible mediator of the enhanced cell growth of MCF7 in response to stromal cell CM. Previous studies have examined the role of chemokine receptors in breast cancer biology. MCF7 and HT-29 (a colon cancer cell line) expressed similar levels of CXCR4, but only MCF7 expressed higher levels of CXCR7, which correlated with enhanced cell growth advantage in response to stromal cell CM. Also, signaling via CXCR7 has been reported to promote cancer cell growth^11^. At a molecular level, CXCR4 binds to CXCL12/SDF-1, whereas CXCR7 homodimer and CXCR7/CXCR4 heterodimer bind to both CXCL11 and CXCL12/SDF-1. We have previously reported that stromal cells express CXCL12, but not CXCL11^13^. Our loss-of-function studies showed that inhibition of CXCR7, but not CXCR4, was able to abolish the observed increase in cell proliferation in MCF7 cells, suggesting that CXCR7/CXCL12 genetic circuit regulates the growth responses in MCF7 cells.

Our findings have clinical relevance, as shown by our survival analysis of a cohort of cancer patients with different types of cancers. Levels of gene expression of CXCR7 were positively correlated to increased overall mortality across different cancer types. Similar to our findings, a recent meta-analysis demonstrated that high expression of CXCR7 is associated with higher risk of lymph node metastasis (LNM), higher tumor grade, poorer overall survival (OS), and shorter recurrence-free survival, across multiple cancer types^14^. These findings suggest that CXCR7 expression can be employed as a prognostic marker for multiple cancer types. Future longitudinal clinical studies are needed to corroborate the prognostic value of CXCR7 expression and to provide a quantitative estimate of its predictive value. Also, pharmacological targeting of CXCR7 signaling represents a possible approach for breast cancer therapy.

## Conclusion

Our data provide an insight into stromal-derived microenvironmental cues that interact with cancer cells, and identify CXCR7 signaling as an important cancer cell growth-promoting factor and that tumor tissue expression level is an unfavorable prognostic marker in breast and several other cancer types.

## Materials and methods

### Cell lines and culture

Cell lines used in the current study covered a broad spectrum of cancer types, including breast (MDA-MB-231, MDA-MB-468, MCF7), colon (HT-29), head and neck (FaDu), and prostate (PC-3). Tumor cell lines were purchased from CLS Cell Lines Service GmbH (Eppelheim, Germany) or American Type Culture Collection (ATCC; Manassas, VA). The well-characterized telomerized hBMSC cell line (hBMSC-TERT) was used as a model for primary hBMSCs^15^. All cell lines were maintained in Dulbecco’s modified Eagle’s medium (DMEM) supplemented with 4500 mg/l d-glucose, 4 mM l-glutamine, 110 mg/l sodium pyruvate, 10% FBS, 1% penicillin–streptomycin, and nonessential amino acids (NEAA).

### Preparation of conditioned media

Tumor-derived conditioned media (TCM) and hBMSC CM (MCM) were prepared using same protocol. Tumor cells (MDA-MB-231, MDA-MB-468, MCF7; HT-29; FaDu; and PC-3) and hBMSCs were seeded individually in six-well plates at 1 × 10^6^/well (4 ml total) in DMEM supplemented with 10% FBS, 1% NEAA, and 1% penicillin/streptomycin, and incubated at 37 °C and 5% CO_2_. At 70–80% confluence, the cells were washed with serum-free DMEM, and the media were then replaced with fresh DMEM media supplemented with 1% FBS, 1% NEAA, and 1% penicillin/ streptomycin, and incubated at 37 °C and 5% CO_2_. After 72 h, the media were collected and centrifuged at 300 × *g* for 10 min to remove any cellular content and debris, and were then stored at −80 °C until used.

### Light microscopy

Tumor cells were cultured on chamber slides (Lab-Tek chamber slides; Nunc, Naperville, IL) at a density of 0.06 × 10^6^/ml in MCM, TCM, or FM, and then incubated at 37 °C under 5% CO_2_. On day 6, cells were fixed in 4% paraformaldehyde followed by hematoxylin and eosin (H&E) staining. Stained slides were mounted, covered, and scanned using ScanScope (×20 or ×60 magnification). The Nikon^®^ ECLIPSE Ti-U inverted microscope was used to image the unstained tumor cells' confluency (×10 magnification).

### Measurement of cell viability

Tumor cells were seeded in 96-well plates at a density of 6 × 10^3^/100 μl/well. Each cell line was seeded on day 0 in fresh media (FM), TCM, or MCM, and was incubated at 37 °C and 5% CO_2_ (each experiment was done at least three times). Cell viability was assessed on day 2, 4, and 6 using the alamarBlue assay. At the indicated time points, 10 μl of alamarBlue reagent (BUF012B; AbD Serotec, Kidlington, UK) was added to each well and was incubated for 3 h at 37 °C. Subsequently, plates were read using BioTek Synergy 2 Multi-Mode plate reader (BioTek, Winooski, VT).

### Transwell experiments

Tumor cells were seeded in the lower compartments of a transwell system (0.4 µm pore size, BD Biosciences) at a density of 0.06 × 10^6^/1 ml, while the upper inserts contained hBMSCs, same tumor cell line or FM. Cells under various experimental conditions were incubated at 37 °C and 5% CO_2_. On day 6, cell viability was assessed using alamarBlue as described above.

### Transfection and small-molecule inhibitor experiments

The validated siRNA targeting human CXCR7 (assay ID: 109229) and FAM-labeled scrambled control siRNA (cat no. AM4620) were purchased from Applied Biosystems (Invitrogen, Carlsbad, CA, USA). Transfection was performed using a reverse transfection approach, as described before^16^. Briefly, siRNA at a final concentration of 30 nM was diluted in 50 µl of Opti-MEM (11058-021; Gibco, Carlsbad, CA, USA), and 1 µl of Lipofectamine 2000 (catalogue no. 52758; Invitrogen) was diluted in 50 µl OPTI-MEM. The diluted siRNA and Lipofectamine 2000 were mixed and incubated at ambient temperature for 20 min. Twenty microliters of transfection mixture was added to the tissue culture plate, and subsequently 10,000 cells in 60 μl transfection medium (complete DMEM without antibiotics) were added to each well. Twenty-four hours later, the transfection cocktail was replaced with complete DMEM. Small-molecule inhibitor targeting CXCR4 (WZ811) was purchased from Selleckchem Inc. (Houston, TX) and was used at 1.0 µM.

### Survival analysis and bioinformatics

Kaplan–Meier plots for overall survival in different cancer RNASEQ datasets were conducted as described before^17^. Network analysis was conducted as described before^18^. Gene expression data for MCF7, FaDU, MDA-MB-231, PC-3, HT-29, and MDA-MB-468 from the Cell Line Encyclopedia (CCLE) project were retrieved from the Gene Expression Omnibus (GEO) depository under accession number GSE36133. Data were subsequently analyzed in R as described before^19^.

### Statistical analyses

Statistical analysis and graphing were performed using Microsoft EXCEL 2016 and GraphPad Prism 6.0 software (GraphPad software, San Diego, CA, USA). *P* values were calculated using the two-tailed *t*-test and *p* value < 0.05 was considered significant.

## Supplementary information

Supplementary Table1
